# A Link between the Increase in Electroencephalographic Coherence and Performance Improvement in Operating a Brain-Computer Interface

**DOI:** 10.1155/2015/824175

**Published:** 2015-07-28

**Authors:** Irma Nayeli Angulo-Sherman, David Gutiérrez

**Affiliations:** Centro de Investigación y de Estudios Avanzados (CINVESTAV), Unidad Monterrey, 66600 Apodaca, NL, Mexico

## Abstract

We study the relationship between electroencephalographic (EEG) coherence and accuracy in operating a brain-computer interface (BCI). In our case, the BCI is controlled through motor imagery. Hence, a number of volunteers were trained using different training paradigms: classical visual feedback, auditory stimulation, and functional electrical stimulation (FES). After each training session, the volunteers' accuracy in operating the BCI was assessed, and the event-related coherence (ErCoh) was calculated for all possible combinations of pairs of EEG sensors. After at least four training sessions, we searched for significant differences in accuracy and ErCoh using one-way analysis of variance (ANOVA) and multiple comparison tests. Our results show that there exists a high correlation between an increase in ErCoh and performance improvement, and this effect is mainly localized in the centrofrontal and centroparietal brain regions for the case of our motor imagery task. This result has a direct implication with the development of new techniques to evaluate BCI performance and the process of selecting a feedback modality that better enhances the volunteer's capacity to operate a BCI system.

## 1. Introduction

A brain-computer interface (BCI) is a system that translates measurements of brain activity into signals for controlling an external device [1]. Noninvasive BCIs usually rely on multichannel electroencephalographic (EEG) data, but the performance of such systems depends on the capacity of the user to modulate his/her brain signals. Therefore, BCI users are trained to develop the ability of altering their brain signals at will.

Most training methods for BCI systems involve performing particular cognitive tasks, such as motor imagery (MI), which is known for altering the *μ* (8–12 Hz) and *β* (13–25 Hz) brain rhythms, specifically at the sensorimotor areas of the brain [2]. Furthermore, the user can be provided with feedback during the training stage to indicate to him/her if a sensorimotor rhythm (SMR) modulation is in fact being achieved. Such feedback usually consists of external stimuli which provide information related to the outcome of the BCI system [3].

Visual presentation of stimuli is the most common feedback modality [4] and it is reported to be the sensory input that leads to the largest improvements in commanding BCI systems [5]. However, there are cases when other kinds of feedback are required, so other feedback modalities like auditory [2, 6] and vibrotactile [7, 8] have been previously tested. Nevertheless, the way in which the feedback should be optimally provided is still a matter of discussion. Specifically, it is not well known if feedback should be delivered when the user is achieving SMR modulation (i.e.,* positive* feedback) or when a detectable SMR modulation is not being reached (*negative* feedback).

In a preliminary work (see [9]), we evaluated the performance of MI-BCI systems with vibrotactile or auditory feedback, in either positive or negative modality. Each of these paradigms was compared against visual feedback, and then we determined if any of the modalities provided a better performance in operating the BCI system for various healthy naive volunteers. Our results showed that the feedback modality that provided the best overall performance varies among subjects, so it should be personalized. However, not all the subjects were able to reach a BCI control level, which was considered to be above 70% of accuracy as it has been previously established as the threshold level to achieve an effective communication through a BCI system [10]. Therefore, alternative training paradigms must be implemented and a measure of their effectiveness must be proposed to facilitate training modality selection.

A candidate for such a measure is the EEG coherence, which reflects the level of coupling of brain activity between different brain regions. In particular, the coherence of the *μ*-rhythm is known to be dominant over frontocentral derivations [11]. Before movement onset, the coherence in 10 and 20 Hz increases between the frontal areas and the sensorimotor areas that are contralateral to the performed movement [12]. This increment coincides spatiotemporally with the event-related desynchronization (ERD) associated with movement execution. After the movement onset, the frontocentral coherence becomes symmetrical, and then it decreases to its base level. On the other hand, centroparietal coherence modulation caused by movement performance or sensorial stimulation has also been reported [13]. Before movement or stimulation and, as part of an anticipation mechanism, there is an increase in the coherence in the centroparietal areas that are contralateral to the movement or stimulated body part. The parietal areas of the brain integrate external and internal information in a compatible reference frame and, along with the central cortex, contribute to transforming sensorial information into operative motor commands. Furthermore, in [14], an increase of the coherence before a movement was observed between the supplementary motor area (SMA) and the left parietal area (contralateral to the movement there executed). After the movement onset, the coherence decreased to its original level. Remarkably, no direct coherence between the parietal and the motor cortices was found. Hence, it is inferred that other areas, such as the SMA and the parietal region, are critical for movement preparation for performing complex movements.

In the present work, we study the correlation between the EEG coherence of the SMR and the performance achieved in operating a MI-BCI system. Our aim is to determine if the coherence is suitable for evaluating the effectiveness of different training paradigms. Hence, this paper is organized as follows: Section 2 describes our experiments and the analysis performed on the acquired data, Section 3 presents the main findings for each of the volunteers we tested, Section 4 discusses those findings in the context of their relevance to mental processes, and Section 5 shows our main conclusions and poses future work.

## 2. Methods

### 2.1. Data Acquisition

EEG measurements are obtained using the B-Alert X10 system (http://www.advancedbrainmonitoring.com/), which has nine acquisition channels located at Fz, Cz, POz, F3, F4, C3, C4, P3, and P4 (according to the international 10/20 system), and referenced to linked mastoids. The B-Alert X10 system filters the EEG measurements with a fifth-order bandpass filter that has its cutoff frequencies at 0.1 Hz and 100 Hz. The B-Alert allows for a resolution of ±1000 *μ*V of the EEG data, which is sent wirelessly to the BCI system at a sample rate of 256 Hz.

### 2.2. MI-BCI System

Our system corresponds to the one already implemented in the BCI2000 freeware platform for *μ*-rhythm-based BCI [15]. There, the potential users are taught to operate the BCI system through training sessions consisting of two stages:* stimulus presentation*, in which the modulation of the *μ*-rhythm is exercised, followed by a* cursor task*, where a personalized MI condition is used as control command. More details about these two stages are provided in Sections 2.2.1 and 2.2.2, respectively. The MI conditions we asked our volunteers to perform were, for the case of the study in [9], the imagination of moving the left hand, right hand, both hands, and both feet. For the current study, we implemented two other tasks: the imaginary flexion of the right middle finger and a condition in which the volunteers performed no imagery movement, but instead functional electrical stimulation (FES) was provided. FES was applied using the MP36 data acquisition system from Biopac (http://www.biopac.com/), the* Biopac student lab stimulator* (BSLSTM), a pair of leads, and a pair of electrodes. Thus, in addition to the code interfacing to the B-Alert acquisition system, BCI2000 routines were adjusted to activate the electrical stimulation. This was achieved by delivering a command that triggered the BSLSTM to supply the FES. The electrodes were placed in the right forearm to produce a twitch in the middle finger. In our case, FES served as a reinforcement of the imaginary flexion of the finger by providing a specific representation of the cognitive event.

#### 2.2.1. Stimulus Presentation

During the stimulus presentation phase the user sat in front of a screen that displayed different visual cues to ask the user to perform various cognitive conditions:

- *MI without FES,* in which visual cues commanded the user to imagine moving either the left hand, right hand, both hands, or both feet when an arrow showed up on the screen pointing either left, right, upside, or downside, respectively. Each of these cues were presented for 3 s in random order. Between each cue presentation, the display was cleared out for 2–2.5 s. During this interval, the user remained at rest. Visual cues were presented until five runs of five sequences of each kind of cue had been shown. There was a 10-minute break between each run.
- *MI plus FES*, in which visual cues indicated to the volunteer either to imagine the movement of the right middle finger or to stay still and pay attention to the sensation produced by FES while watching the screen. Each of these states was randomly presented for 3 s. Between these periods there was also a lapse of 2–2.5 s in which the screen went blank to ask the subject to remain at rest. The cue assigned for the MI condition was the word “Imagine,” while for the case of FES the cue was a video showing a right hand with its middle finger twitching as consequence of FES. Moreover, the video sequence was nearly synchronized with the real movement produced on the volunteer, who received FES at 2 Hz and at a comfortable voltage level during the 3 s that the presentation of the FES cue lasted. For our study, the video and FES together had the single purpose of creating a mental image going along with the sensation of the finger flexion. Even though it is out of the scope of our study, the use of FES in a long term training could involve neuroplasticity, as FES has been reported to promote motor relearning through the repetition of specific movements and by promoting cortical reorganization [16]. In this stimulus presentation stage, the visual cues were shown until five runs of ten sequences of each cue were presented. There was a 5-minute break between runs.We have to mention that there was the case of one subject who, instead of being presented a video as the FES cue, was shown the message “Estimule” (Spanish for* stimulate*, as our volunteers were native Spanish speakers) to indicate to the user to pay attention to FES. The difference on the type of visual cue was because this subject was the first in our experimental protocol to receive FES as part of the BCI training. From this subject's experience, we later improved the protocol for other subjects with the video cue hoping that the users could associate FES and MI in a better way, perhaps by creating a mental image of the stimulation.

**Figure 1:**
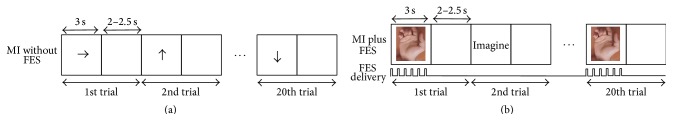
Scheme of a run for the different stimulus presentation modalities: MI without FES (a) and with FES (b).

During the stimulus presentation stage, the number of sequences in the case of FES was larger in order to equalize as much as possible the duration of the run in comparison to the stimulus that did not include FES. All the different stimulus presentation modalities that were previously described are schematized on Figure 1.

The EEG data measured during the stimulus presentation stage for all different cognitive conditions were analyzed with help of the BCI2000 offline tools. The analysis consisted in the calculation of the *r* ^2^ values for all electrodes within the default tool's frequency range of 0–70 Hz for two different cognitive states. However, for the purposes of this study, we only consider those *r* ^2^ values consistent with the expected frequency range of the SMR (i.e., below 25 Hz). Furthermore, the *r* ^2^ values were computed for the MI either of the right hand or of the right middle finger, both compared against the rest state condition. The *r* ^2^ value represents the fraction of the variance of a measurement (explained sum of squares/total sum of squares) which provides a sense of the spectral amplitude, in a particular frequency and a specific scalp location, that can be predicted from a cognitive state [17]. *r* ^2^ is a measure of how well two cognitive conditions can be discriminated; then the higher its value is, the more discernible the states are. Based on that, we selected the frequency (denoted by *f*_SMR_) and the EEG sensor with higher *r* ^2^ value as a personalized feature for BCI control. As an example, the selected frequencies for BCI control during the last session of Subjects 1–7 were 9, 19, 23, 11, 12, 11, and 23 Hz, respectively. Note that these parameters were verified every session and adjusted if necessary. However, we always found that the selected parameters were consistent with the SMR properties because *f*_SMR_ was within the frequency range of *μ* and *β* brain rhythms. In addition, the chosen EEG channel was for all cases C3, which is positioned over the sensorimotor cortex, in particular at the left side of the scalp, contralateral to the imagined movement, all in accordance with the region where the brain activity is expected to occur.

#### 2.2.2. Cursor Task

In this stage, the personalized MI cognitive task was used to control the movement of a cursor on the screen. Hence, runs of 32 independent attempts (trials) of such control were completed in order to determine the performance of each user in operating the BCI. As an aid for the control, our volunteers received different types of feedback at this stage.

(i) *Visual feedback*: during each trial, subjects sat in front of a computer screen where a target appeared represented by a vertical bar that could be positioned at the top or bottom half of the right side of the screen. Afterwards, a ball (also called a* cursor*) showed up on the left edge of the screen, moving horizontally at constant speed and with its vertical motion being controlled by the magnitude of the detected SMR modulation at the EEG sensor and *f*_SMR_ previously selected. The detection of MI moved the cursor upward, while a resting state displaced the cursor downward. Thus, the goal in the trial was to hit the target, which required the control of the vertical movement of the cursor.
(ii) *Positive or negative auditory feedback*: in each trial, the subject was placed in front of the screen and the target was still presented, but not the cursor. Then, the correct or incorrect displacement of the cursor relative to the target was suggested by a constant sound tone of 300 Hz. For positive feedback, the tone was presented only when the hidden cursor moved vertically towards the objective, so the tone indicated that an adequate SMR modulation was being achieved. On the contrary, in the case of negative feedback, the auditory stimuli occurred only when the cursor went against the direction of the objective, warning the user about the lack of control of the cursor.

**Figure 2:**
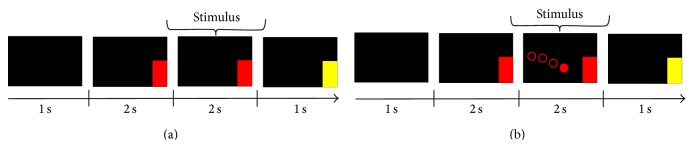
Time sequence of a testing trial for the cases of auditory feedback (a) and visual feedback (b).

**Figure 3:**
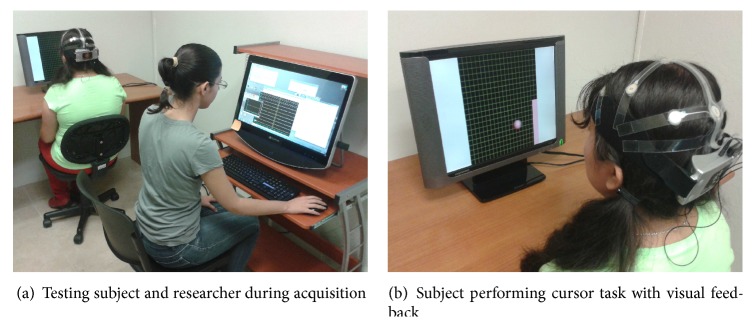
Experimental arrangement during sessions (same as in [9]).

The following sequence for each evaluation trial was used in our experiments: The target was shown for 2 s; then feedback was presented for 2 s. The trial ended with a postfeedback resting period of 1 s after which a new trial was started. Also, the color of the target changed to yellow when the cursor hit it to indicate that a successful trial was completed. The sequence of events for each feedback modality is presented in Figure 2, while Figure 3 shows the experimental setup for the cursor task. In all runs, the number of times the subject hit the target was recorded. From this point on, we will refer to the percentage of hits as the* accuracy*, which will be used as a measure of the BCI performance for all users and all training paradigms.

### 2.3. Subjects

Seven volunteers (three male and four female, aged 20 to 32 years, mean age 26.3 ± 4.2) were trained to achieve SMR modulation in order to control the MI-BCI system described in Section 2.2. Subjects 1, 2, 3, and 4 were part of the experiments in [9], while the rest were naive in terms of exposure to a BCI system.

Subjects 1–4 used the MI of their right hand to command the BCI system in [9]. There, they trained with different types of feedback during the cursor task: in chronological order, Subject 1 trained with visual feedback (denoted from now on simply by V), positive auditory feedback (or PA), and negative auditory feedback (NA) training modalities; Subject 2 trained with PA and V modalities; Subjects 3 and 4 trained with NA and V training paradigms. In [9], the users participated in up to seven sessions of up to seven runs for each training with 10-minute breaks between runs. Furthermore, the stimulus phase was presented only up to the fourth session because this stage was used to establish the best features for BCI control, that is, to determine the best MI among each hand, both hands, and both feet. For all these volunteers, the best feature turned out to be the imagery movement of the right hand. For this reason, the right hand was selected as the base position for providing FES in a new training scheme, but because our FES equipment can only supply a limited voltage to a small area, moving the right middle finger was chosen as the best option for motor imagery. This way, we expected brain activity to be detected over the same EEG electrode.

Our results in [9] suggested that four training sessions were enough to determine if a training paradigm was effective. For this reason, in this follow-up study, Subjects 2 to 7 were subjected to four sessions of a training paradigm that included FES during the stimulus presentation, as well as visual feedback in the cursor task stage (except for Subject 6, who completed five sessions). Subject 5 was the one who received FES training that did not include video, as we previously mentioned in Section 2.2.1.

In all cases, the schedule of the training sessions varied, but we tried to keep the same training pace in the case a volunteer was being evaluated for more than one modality. In average, it took four weeks and a half for our volunteers to complete both the stimulus presentation and cursor task for a training modality.

### 2.4. Data Analysis

The next sections explain the process we followed to analyze the EEG data collected for all our volunteers (including the data previously acquired in [9]).

#### 2.4.1. EEG Coherence

The coherence is a measure of the degree of correlation of the spectral power in a specified bandwidth between two signals. In particular, the coherence of the EEG provides information about the connectivity of the brain and it indicates when different brain regions communicate with each other to perform cognitive tasks [18]. High coherence means that there is a large degree of communication between different brain regions. On the contrary, a low coherence indicates a relative independence between the different brain locations. In our study, the computation of the coherence is based on a method similar to the one used in [19], where the confidence level of the coherence is assessed for all possible pairs of sensors at a given frequency. In our case, the coherence was computed for frequencies at values *f*_SMR_ − 1, *f*_SMR_, and *f*_SMR_ + 1 Hz, as they are within the range BCI2000 considers for SMR modulation. Furthermore, the coherence was calculated for all $\left(\begin{array}{c} 9\\ 2 \end{array}\right)=36$ combinations of sensors, but we only considered in the computation the portion of the EEG signals corresponding to the 2 s during the cursor task where feedback was presented. In all cases, the value of the coherence and the threshold for the coherence to be significant were stored. Then, those signals for which the coherence was below the significance threshold (i.e., the coherence whose confidence was not enough to be regarded as indicative of connectivity) were discarded in following calculations.

Given the fact that a volunteer is either performing a MI or at rest during the feedback presentation in the cursor task, we subtracted the mean value of the coherence at rest to those when performing a MI. We will refer to this last calculation as the* event-related coherence*, or ErCoh, as suggested in [13]. Therefore, as final result of these calculations, we end up with the values of ErCoh for selected pairs of sensors. For the following analysis of these data, ErCoh values from the frequencies *f*_SMR_ − 1, *f*_SMR_, and *f*_SMR_ + 1 Hz were considered part of a single bin, so no distinction between the frequencies that composed the bin was made.

#### 2.4.2. ANOVA Tests

We performed one-way analysis of variance (ANOVA) tests for each subject and for each training paradigm to detect significant changes in ErCoh through different sessions. Also, we performed ANOVA tests for each subject and training modality to evaluate the effect of the number of sessions on the BCI accuracy. In case significant differences between the means (in either ErCoh or accuracy) were found by the corresponding ANOVA test, we performed multiple comparison tests using the Tukey-Kramer method to determine which sessions could be accounted for those differences. All tests were performed using the statistical tools of MATLAB (http://www.mathworks.com/) and at the *α* = 0.05 significance level.

There is reason to believe that improvements on the SMR modulation skills involve some changes on the neural activity that are reflected as an increment on the coherence [20, 21]; then our goal is to determine if such improvement will be reflected as an increase of accuracy. Hence, we looked for trends in ErCoh and accuracy across each training with help of the results from the multiple comparison tests. In particular, an incremental trend was determined if ErCoh or accuracy of the last two or more sessions had a higher value in comparison to the first session. For the cases in which there was an increment in ErCoh, the correlation (denoted by *r*_ca_) between the mean values of ErCoh and accuracy was calculated.

## 3. Results

After evaluating the increments on ErCoh for all subjects with their respective training paradigms, it was found that differences on ErCoh were mainly located at the contralateral centroparietal and centrofrontal regions. Therefore, the results that are presented in this section are focused on those regions for simplicity.

**Figure 4:**
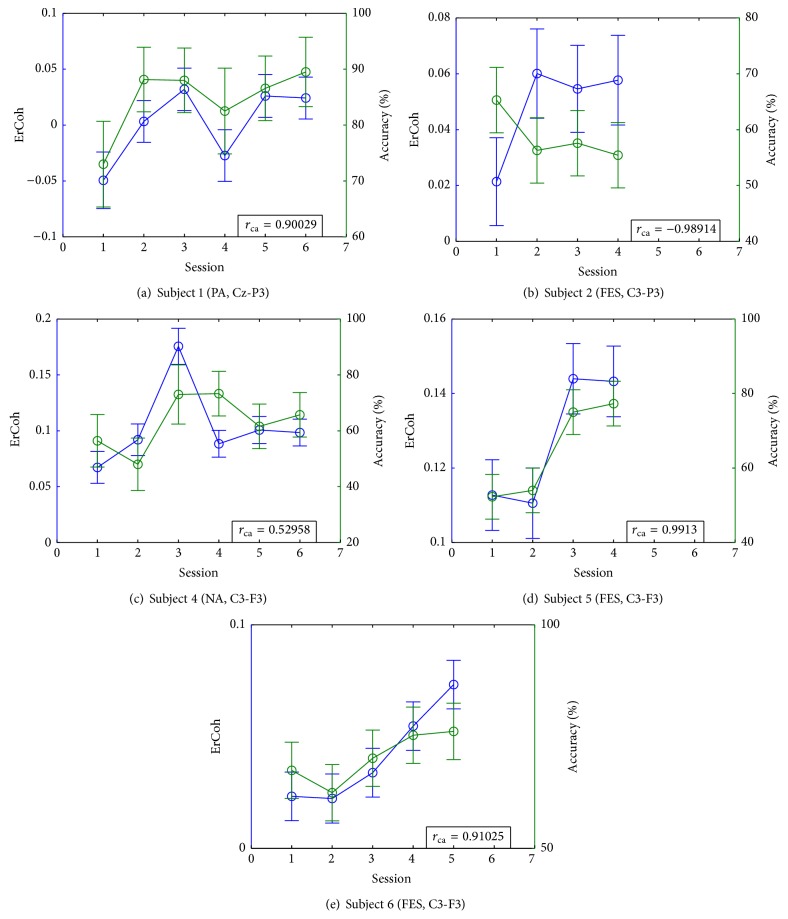
Accuracy and ErCoh (average among trials, error bars indicate the confidence interval) for the cases in which subjects showed an increment on ErCoh in the contralateral centrofrontal or centroparietal areas.

**Figure 5:**
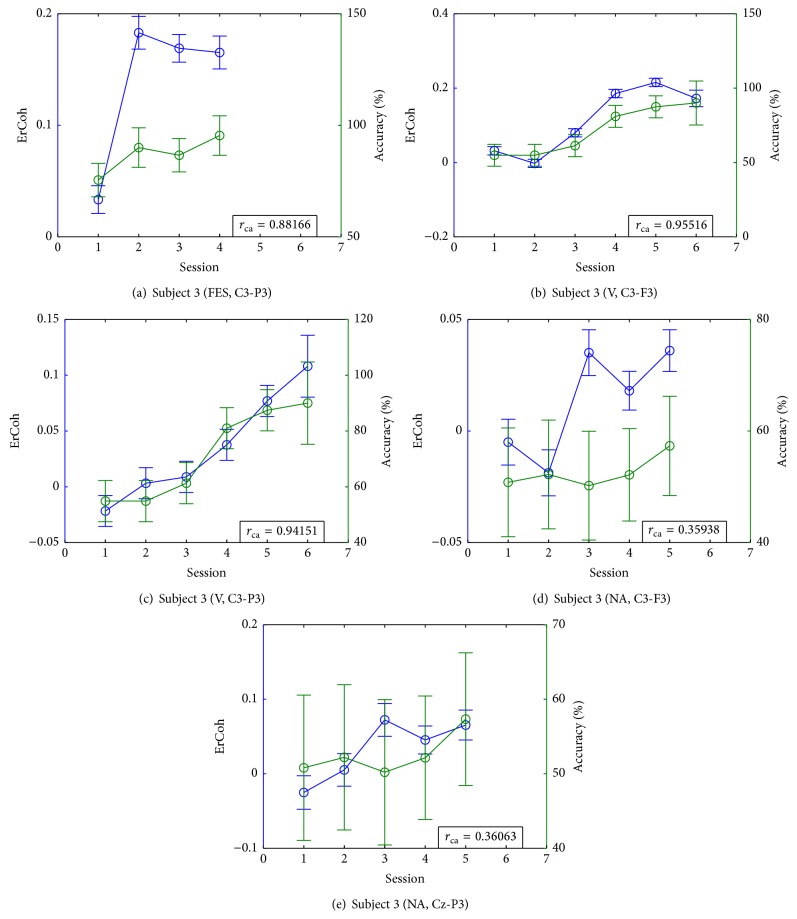
Accuracy and ErCoh (average among trials, error bars indicate the confidence interval) for the cases in which Subject 3 showed an increment on ErCoh in the contralateral centrofrontal or centroparietal areas.

For each subject, Figures 4 and 5 show the accuracy and ErCoh in the cases where centrofrontal or centroparietal incremental trends on those values were found, as well as the correlation *r*_ca_ between the mean ErCoh and the mean accuracy. The corresponding statistics comparing ErCoh and accuracy through training sessions for the same cases are presented on Table 1. There, the session number for a particular training is referred to as S1, S2, and so on. Next, we explain the results for each subject in further detail.

### 3.1. Subject 1

Based on our criteria for finding incremental trends in the measurements, Subject 1 showed a significant increase on ErCoh at the pair of sensors Cz-P3 when training with PA feedback. This is shown on Table 1(a), where the comparison between sessions revealed that the second, third, fifth, and sixth sessions had a significantly higher coherence than the first session. From this point on, such fact will be described with the following notation: S2, S3, S5, S6 > S1. Anyhow, S4 had a lower coherence than S3. Note that these differences match those found on the accuracy. This fact can be inferred by comparing the results on the multiple comparison tests in Table 1(a) with those on Table 1(b), as well as from Figure 4(a); hence, high correlation between those two measurements is expected. The combination Cz-P3 involves the supplementary motor area (SMA) and the parietal cortex that is contralateral to the imagined movement. The increase on the coherence in this area has been associated with the preparation for performing complex movements (as an auxiliary network that involves the motor and parietal regions). Then, for this subject, activity on this network seems to become enhanced with SMR modulation training.

### 3.2. Subject 2

In the case of Subject 2, Figure 4(b) and Table 1(a) show that ErCoh increased from S2 in C3-P3 with FES training. This increasing trend was maintained until the last session. Nevertheless, this subject showed no significant changes on accuracy, as observed in Table 1(b). However, there was a high negative correlation between the ErCoh and performance. This could be explained by mere randomness given that the subject was never in control of the BCI (accuracy levels close to 50%).

### 3.3. Subject 3

Subject 3 shows significant increments on ErCoh through sessions for all types of training, although an increase on performance cannot be observed on all training paradigms. For FES training, there was a significant increase in ErCoh on C3-P3 starting on S2 that is sustained until the last session (see Figure 5(a)). Also, the last session had significantly higher accuracy in comparison to the first session of the training, after which a performance higher than 70% was achieved. The results of the statistical analysis can be seen in Tables 1(a) and 1(b), along with the results of other training paradigms for this subject. ErCoh at C3-P3 is highly correlated with the accuracy during the training. This could be an indication of an increase in the functionality between the motor and the parietal cortices, which is reflected as an increase of ErCoh through sessions. This way, FES training could be helping the subject to produce an adequate SMR modulation to control the BCI, since the coherence increment in the centroparietal area that is contralateral to the produced movement is associated with a better transformation of sensorial and internal information into a movement. This very similar relationship could be observed during movement imagery.

For the case of V training, there was a significant increase on both ErCoh and accuracy on C3-F3 and C3-P3 that is clearly shown in Figures 5(b) and 5(c), respectively. The increment on both measures is highly correlated. The increase on ErCoh over the centrofrontal region in this case serves as an additional network associated with SMR, and its development could have helped the subject to gain control of the MI-BCI.

On the other hand, NA training exhibits increasing ErCoh through sessions at C3-F3 and Cz-P3, but no significant differences on accuracy, as shown in Figures 5(d) and 5(e). Then, there was low correlation between ErCoh and BCI performances for this training paradigm. In spite of this, it is important to consider that the accuracy with NA was close to 50%. Thus, these observed variations on BCI performance are more related to randomness rather than being caused by the subject. Hence, it is not surprising that there was a low correlation of this measure with ErCoh. Nevertheless, it is clear that the effects of the two pairs of sensors where an increase in ErCoh was detected were not enough for the subject to achieve control over the BCI. In general, this could say that even if there are multiple brain networks related to BCI control, not all of them have the same impact on BCI performance for a particular subject.

### 3.4. Subject 4

Figure 4(c) shows the accuracy and ErCoh for Subject 4 with NA. There, and in Table 1(a), it can be seen that there is a significant increase in ErCoh at C3-F3. The accuracy of this subject at the last session was not higher in comparison to S1. However, S3, S4, and S6 had a higher accuracy in relation to S2, as shown in Table 1(b). The correlation between ErCoh and accuracy is not high, although it needs to be noted that the performance is low and it has a great variability too. Hence, it is difficult to relate coherence and accuracy for this case.

### 3.5. Subject 5

The results for Subject 5 are shown in Figure 4(d), and Tables 1(a) and 1(b) for the case of FES at C3-F3. These results show a significant increase of ErCoh through different sessions, as well as a higher accuracy at the last two sessions compared to the first two sessions. At the end, the subject reached an accuracy above 70%. Also, there was high correlation between ErCoh and accuracy. This could be associated with a relationship between SMR modulation and BCI performance. The increase appears on a location important for SMR, which is a desirable condition to reach BCI control.

### 3.6. Subject 6

Figure 4(e) shows an increase on ErCoh for Subject 6 in the case of FES at C3-F3. Furthermore, Table 1(a) supports this by showing that the last two sessions have a significantly higher ErCoh in comparison to the first two sessions. There was a high correlation between ErCoh and accuracy, and this last one reaches values above 70% by the end of the training. Even though the statistical analysis on Table 1(b) shows that the last two sessions have significantly higher performance compared to S2, there was no incremental trend on performance because the accuracy of S1 was not significantly different to the last two sessions. The high value of *r*_ca_ could be explained by the fact that the *μ*-rhythm is found mainly at frontocentral regions, and its activity augments the coherence. Thus, a higher ErCoh could mean that there is a greater difference between the two detection conditions (MI and rest) for BCI control, which would imply a better discrimination of the cognitive events, thus better performance.

Note that not all increments in coherence lead to a high correlation with BCI performance. In spite of this, for all subjects (except for Subject 2) there was at least a pair of channels for which coherence and accuracy were highly correlated for at least one training. These pairs were located at frontocentral or centroparietal areas. A similar behaviour was found also for Subject 7. However, this subject did not show an incremental trend of ErCoh in any pair of electrodes. This specific case is described next.

### 3.7. Subject 7

**Figure 6:**
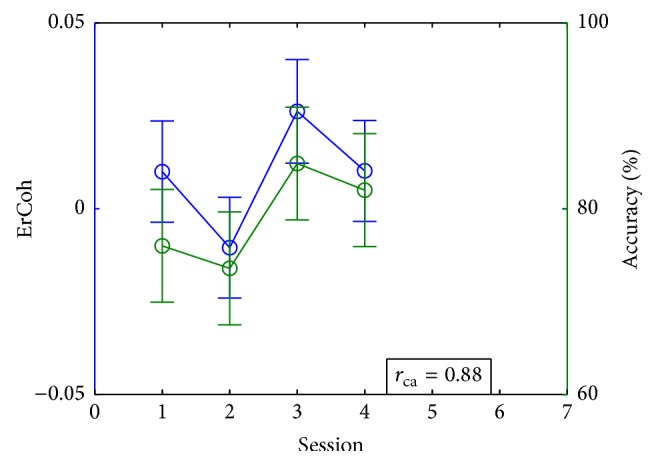
Accuracy and ErCoh (average among trials, error bars indicate the confidence interval) for Subject 7 with FES training at C3-P3.

The results for Subject 7 are shown in Figure 6 and Table 2 for the case of FES at C3-P3. These results show that there was no statistically significant difference in accuracy for different sessions. Even though we found a significant difference between ErCoh in the second and third sessions, it was not associated with an incremental trend through training. Nevertheless, this user achieved a performance above 70% since the first session, so the subject could always control the BCI system. Perhaps accuracy variations between sessions cannot be directly associated with the training scheme, but there was a high correlation between ErCoh and accuracy. This can be due to the fact that ErCoh from the contralateral centroparietal area is associated with the integration of sensorial stimuli to produce appropriate motor signals, so it may be related to the performance of the MI-BCI. Moreover, the results of this user show that it is not necessary to have an ErCoh increment in order to find a correlation between it and accuracy, which makes sense since BCI control was achieved by the user from the start.

## 4. Discussion

So far our results show that six out of seven subjects exhibit an increase in coherence of their SMR compared to the rest state (as measured by ErCoh) for some type of training at the centrofrontal or centroparietal contralateral sites. Among these subjects, four out of six volunteers presented an increase in ErCoh through training sessions that was highly correlated with BCI performance. The pairs of channels that showed such high correlation have different effects on movement execution according to the literature: centrofrontal coherence is directly associated with SMR activity, while centroparietal coherence is related to the integration of exteroceptive and proprioceptive information to produce suitable motor commands. On the other hand, the coherence of Cz-P3 suggests the existence of an auxiliary network involving SMA and the parietal region. The action of such network may be preparing the subject for complex movements. These results were previously reported for SMR of actually performed movements, but our results seem to verify a similar phenomenon for movement imagery.

It is important to consider a limitation when finding the correlation between mean accuracy and mean ErCoh, which is when the accuracy is near chance level, as variations on accuracy could be produced more by the inherent randomness of the BCI system rather than by the user. On the other hand, there is the possibility that ErCoh could be a better measure of the effect of training for a MI-BCI rather than the accuracy when there is poor BCI control. However, it would be difficult to evaluate this considering that the reference (and more practical way) to evaluate the effectiveness of these kinds of systems is the accuracy. Thus, the detection of the most relevant pair of electrodes of the centrofrontal and centroparietal channels that affect the performance would be useful to augment the information that could be used to evaluate the effect of a specific training. Furthermore, the ErCoh of these regions could be trained within the BCI design as a possible way to improve the performance for a MI-BCI. Nevertheless, not all the users seem to have the same outcome on the BCI performance with an increasing ErCoh at the same brain region. For this reason, in order to use the ErCoh as an alternative or complimentary measure of the accuracy, it would be necessary to establish a method to identify the pairs of electrodes that have an impact on the BCI performance for using such pairs as a reference for the BCI evaluation.

Moreover, it would be important to evaluate the coherence using more electrodes and analyzing other bands in the future. For example, in [22] a low performance in tracking a target on a screen through a visuomotor task was related to an increment in the centrofrontal coherence of the alpha band. Hence, a more integral understanding of the cognitive processes could be used to develop new training strategies for BCI.

In addition, particular effects of different training schemes on the coherence must be evaluated and associated with BCI performance. Because our study just looked for a relationship between SMR coherence and accuracy, finding ErCoh differences as a function of the various training schemes was not within the scope of the study. Hence, effects on ErCoh due to FES training, either before or during a BCI task, require further study. FES was integrated in BCI training with the aim of providing a specific representation that could facilitate motor imagery. From a qualitative point of view, this strategy seemed to provide satisfactory results for most of our subjects, but further confirmation with diverse training strategies should be obtained in order to effectively assess the effects of FES. This evaluation could be done with a methodology similar to the one we proposed in [9].

## 5. Concluding Remarks

We proposed an evaluation procedure for different BCI training paradigms which is based on a measure of the difference in SMR coherence between motor imaginery and rest state, as well as changes on the accuracy. Also, we computed the correlation between these two measurements in a search for an insight about mental processes that enhance performance. In our experiments, six out of seven subjects showed an increase of such difference of coherence in the centroparietal or the centrofrontal regions. Out of these six volunteers, four presented a high positive correlation of their ErCoh with their respective performance. The centrofrontal and the centroparietal areas involving the contralateral regions to the movement of a body part appear to be associated with the SMR activity and the production of a satisfactory motor activity based on external and internal information with respect to the body, respectively.

Finally, we introduced FES as an aid in the process of training. From that we concluded that FES training was successful, but the mechanisms by which it helped to enhance performance still need to be studied.

Future work in this area would include developing a method for a blind selection of the electrodes that affect BCI performance (instead of evaluating all possible combinations) in order to use ErCoh as an alternative or complimentary measure of the accuracy in MI-BCIs.

## Figures and Tables

**Table:** (a) ErCoh analysis

Subject	Training	Compared sessions	Pair of electrodes	*F*	*p* value	Multiple comparisons
1	PA	S1–S6	Cz-P3	8.88	2.74 × 10^−8^	S2, S3, S5, S6 > S1; S3, S5, S6 > S4

2	FES	S1–S4	C3-P3	97.73	0.0045	S2, S3, S4 > S1

3	FES	S1–S4	C3-P3	3.74	2.52 × 10^−56^	S2, S3, S4 > S1
	V	S1–S6	C3-F3	247.35	1.54 × 10^−200^	S5 > S4, S6 > S3 > S1 > S2
			C3-P3	32.54	8.04 × 10^−32^	S5, S6 > S4 > S3, S2, S1; S3 > S1
	NA	S1–S5	C3-F3	21.91	1.56 × 10^−17^	S3, S4, S5 > S1, S2
			Cz-P3	13.54	8.60 × 10^−11^	S3, S5 > S1, S2; S4 > S1

4	NA	S1–S6	C3-F3	23.51	6.96 × 10^−23^	S5, S4 > S1; S3 > S1, S2, S4, S5, S6

5	FES	S1–S4	C3-F3	12.57	4.17 × 10^−8^	S3, S4 > S1, S2

6	FES	S1–S5	C3-F3	15.23	3.09 × 10^−12^	S4, S5 > S1, S2; S5 > S3

**Table:** (b) Accuracy analysis

Subject	Training	Compared sessions	*F*	*p* value	Multiple comparisons
1	PA	S1–S6	3.44	0.0146	S2, S3, S5, S6 > S1

2	FES	S1–S4	2.27	0.1060	

3	FES	S1–S4	4.38	0.0159	S4 > S1
	V	S1–S6	18.08	2.21 × 10^−8^	S4, S5, S6 > S1, S2, S3
	NA	S1–S5	0.4174	0.7944	

4	NA	S1–S6	5.36	0.0013	S3, S4, S6 > S2

5	FES	S1–S4	18.81	1.71 × 10^−6^	S3, S4 > S1, S2

6	FES	S1–S5	3.43	0.0200	S4, S5 > S2

**Table 2:** Results of ANOVA and multiple comparison tests for Subject 7 with a FES training scheme for accuracy and ErCoh at C3-P3.

Subject	Compared sessions	Tested variable	*F*	*p* value	Multiple comparisons
7	S1–S4	ErCoh	3.97	0.0079	S3 > S2
		Accuracy	2.81	0.0612	
